# Band-like arrangement of taste-like sensory cells at the gastric groove: evidence for paracrine communication

**DOI:** 10.3389/fphys.2013.00058

**Published:** 2013-04-02

**Authors:** Julia Anna-Maria Eberle, Patric Richter, Patricia Widmayer, Vladimir Chubanov, Thomas Gudermann, Heinz Breer

**Affiliations:** ^1^Institute of Physiology, University of HohenheimStuttgart, Germany; ^2^Walther-Straub-Institute of Pharmacology and Toxicology, University of MunichMunich, Germany

**Keywords:** gastric groove, brush cells, chemosensory cells, NOS, ChAT

## Abstract

The discovery of taste-related elements within the gastrointestinal tract has led to a growing interest in the mechanisms and physiological significance of chemosensory monitoring of chymus composition. Previous work suggests that brush cells located in the “gastric groove,” which parallels the “limiting ridge,” a structure in rodents that divides the fundus from the corpus, are candidate sensory cells. A novel sectioning technique revealed that these cells are arranged in a palisade-like manner forming a band which borders the whole length of the corpus epithelium. Using transgenic PLCβ2 promoter-GFP mice and specific antibodies, we have demonstrated that most of these cells express gustducin, PLCβ2, and TRPM5; typical signaling proteins of gustatory sensory “type II” cells. These molecular features strongly suggest that the cells may be capable of sensing nutrient or non-nutrient constituents of the ingested food. Since there is no evidence that brush cells are endocrine cells, attempts were made to explore how such putative chemosensory cells might transmit the information to “effector” cells. It was found that most of the cells express the neuronal nitric oxide synthase (NOS) suggesting some paracrine interaction with adjacent cells. Moreover, they also express choline acetyltransferase (ChAT) as well as the vesicular protein SNAP25, indicating the potential for cholinergic transmission, possibly with subjacent enteric nerve fibers.

## Introduction

The adjustment of digestive processes of the gastrointestinal tract according to the amount and composition of the ingested food requires a precise monitoring of luminal composition. There is growing evidence that this is accomplished by a chemosensory system of the alimentary tract, which is related to the gustatory system. Several studies have demonstrated that gustducin-expressing cells are present in the murine gastric mucosa which were considered as candidate chemosensory cells (Höfer et al., 1996; Hass et al., 2007). During the course of these studies it was noticed that numerous gustducin-positive cells are segregated in the so-called “gastric groove” underneath a tissue fold, the “limiting ridge.” This characteristic structure lines the entire fundus/corpus border of the stomach as well as the esophagal orifice (Wattel and Geuze, 1978; Luciano and Reale, 1992). In previous studies only relatively small segments of this region were analyzed and it remained unclear how gustducin-positive, putative chemosensory cells were arranged along the course of the “gastric groove.” Moreover, although the expression of the gustatory G-protein gustducin suggests some relatedness to taste cells, further molecular phenotyping is required to explore whether the cells express other essential elements of the gustatory signaling cascade in “type II” taste cells. In the present study we set out to employ a newly established sectioning technique to examine the arrangement of putative chemosensory cells in the “gastric groove” along the course of the “limiting ridge”. Furthermore, the availability of transgenic PLCβ 2 promoter-GFP mice in combination with improved immunohistochemical approaches permitted visualization of the expression of gustducin, PLCβ2 and TRPM5, key elements of the molecular machinery for sensing nutrients in the gustatory system. In the taste sensory cells activation of the G-protein coupled receptor elicits a second messenger cascade mediated by the characteristic phospholipase C-subtype, PLCβ 2 (Rössler et al., 1998; Margolskee, 2002; Montmayeur and Matsunami, 2002; Medler and Kinnamon, 2004). In addition, attempts were made to explore which mechanisms may be involved to convey the information onto adjacent cells, which could either be closely associated endocrine cells (Hass et al., 2007) or nerve fibers.

## Materials and methods

### Mice

Analyses were performed with wild type mouse strains C57/BL6J purchased from Charles River (Sulzfeld, Germany). In addition a previously described transgenic mouse line was used, which expresses GFP under the control of the PLCβ2 promoter (Kim et al., 2006). Animals were fed with standard laboratory chow *ad libitum* and had free access to water. All experiments comply with the Principles of Animal Care, publication no. 85–23, revised 1985, of the National Institutes of Health and with the current laws of Germany. For tissue preparations animals were killed by cervical dislocation and subsequent decapitation. Prior to perfusion animals were killed by inhalation of lethal doses of carbon dioxide delivered by a compressed gas cylinder.

### RNA isolation and cDNA synthesis

Total RNA was isolated from dissected tissue preparations of the stomach compartments with a Nucleo Spin RNA kit (Macherey-Nagel, Düren, Germany) according to the manufacturer's protocol. To ensure the complete removal of DNA, a DNase digestion (DNaseI, LifeTechnologies, Carlsbad, CA, USA) step was included. Subsequently, 1.0 μg total RNA was reversely transcribed using oligo(dT) primers and SuperScript III Reverse Transcriptase (RT; Invitrogen, Carlsbad, CA, USA). RNA integrity of each sample was controlled by the amplification of the housekeeping gene for the ribosomal protein L8 (rpl8) with intron spanning primers to verify the DNA removal.

### Reverse transcriptase polymerase chain reaction (RT-PCR)

RT-PCR amplification was conducted by using normalized cDNA from different tissues of the stomach compartments. PCR amplifications were performed with the following primer combinations:

ChAT forward, 5′-GTA TGC CTG GAT GGT CCA GGC AC-3′; ChAT reverse, 5′-GTA TGC CTG GAT GGT CCA GGC AC-3′; NOS1 forward, 5′-GCT GCA GCA GTT CGC CTC CCT GG-3′; NOS1 reverse, 5′-CAG ACT CGG CCA GCT GTT CCT GC-3′; NOS2 forward, 5′-CCA GCA TGT ACC CTC AGT TCT GCG-3′; NOS2 reverse, 5′-CAA TCC ACA ACT CGC TCC AAG A-3′; NOS3 forward, 5′-CTG CTG CCC GAG ATA TCT TCA GC-3′; NOS3 reverse, 5′-TTT GCT GCT CTG TAG GTT TTC CA-3′.

RT-PCR was carried out using High Fidelity PCR Enzyme Mix (Fermentas, St. Leon-Rot, Germany) and a Peltier PTC-200 thermo cycler (MJ Research). For amplification of choline acetyltransferase (ChAT) the following PCR cycling profile was used: One cycle: 4 min at 94°C, 40 cycles: 30 s at 94°C, 40 s at 65°C, 90 s at 72°C; and one cycle: 5 min at 72°C.

Amplicons for NOS isoforms were obtained using the following PCR cycling profile:

One cycle: 4 min at 97°C, 40 cycles: 30 s at 97°C, 40 s at 68°C, 90 s at 72°C; and one cycle: 3 min at 72°C.

PCR products were run on a 1% agarose gel containing EtdBr. Amplification of a 204 bp fragment from mouse housekeeping control gene ribosomal protein l8 (rpl8) was used as control to confirm equal quality and quantity of the cDNA preparations. PCR products for ChAT were subsequently cloned into pGem-T (Promega, Madison, WI, USA) and subjected to sequence analysis in an ABI PRISM 310 Genetic Analyzer (Applied Biosystems, Foster City, CA, USA).

### Tissue preparation

For *in situ* hybridization, the stomachs of adult mice were dissected in 1× phosphate-buffered saline (PBS: 0.85% NaCl, 1.4 mM KH2PO4, 8 mM Na2HPO4, pH 7.4), embedded in Leica OCT Cryocompound “tissue-freezing medium” (Leica Microsystems, Bensheim, Germany) and quickly frozen on dry ice. Sections (8 μm) were cut on a CM3000 cryostat (Leica Microsystems, Bensheim, Germany) and adhered to Superfrost Plus microslides (Menzel Gläser, Braunschweig, Germany).

For immunohistochemistry, stomachs of adult mice were dissected in 1× PBS and fixed as described below.

For immunoreactivity to CK18, TRPM5, PLCβ 2, GFP, gustducin, and NCAM, tissue was fixed in 4% paraformaldehyde (in 150 mM phosphate buffer, pH 7.4) for 30 min to 2.5 h at 4°C.

For immunoreactivity to NOS1 and ChAT mice were gassed with CO_2_ and perfused via the left heart ventricle with 1× PBS followed by 4% ice-cold paraformaldehyde. After perfusion the tissue was fixed in the same fixative for 24 h.

Immunoreactivity for ChAT was also achieved by perfusion via the left heart ventricle with 1× PBS followed by 4% ice-cold paraformaldehyde with 0.1% glutaraldehyde (in 150 mM phosphate buffer, pH 7.4). For double-labeling experiments with ChAT and SNAP25 antibodies, the stomach was fixed after perfusion for 1 h in 4% paraformaldehyde and for further 22 h in 1:1 fixative:1× PBS.

After fixation the tissue was cryoprotected by incubation in 25% sucrose overnight at 4°C. Finally, the tissue was embedded in Tissue-Freezing Medium and quickly frozen on dry ice or liquid nitrogen. Cryosections (4–8 μm) were generated using a CM3050S cryostat (Leica Microsystems, Bensheim, Germany) and adhered to Superfrost Plus microscope slides (Menzel Gläser, Braunschweig, Germany).

### *In situ* hybridization

The T7/SP6 RNA transcription system (Roche Diagnostics, Mannheim, Germany) was used, as recommended by the manufacturer, to generate digoxigenin-labeled antisense riboprobes from partial cDNA clones in pGem-T plasmids encoding *Mus musculus* ChAT (Genbank accession number NM_009891.2, positions 1171-2263). The corresponding sense riboprobes were generated to serve as a negative control. Resultant RNA probes were subjected to partial alkaline hydrolysis, as described by Angerer and Angerer (1992), to produce fragments of ~200–250 base pairs in length. Conditions for *in situ* hybridization were as described previously (Hass et al., 2010). Sections were mounted in MOWIOL [10% polyvinyl-alcohol 4-88 (Sigma), 20% glycerol in 1× PBS].

### Immunohistochemistry

Cryosections (4–8 μm) were air-dried, rinsed in 1× PBS for 10 min at room temperature and blocked in 0.3% Triton X-100 in 1× PBS containing either 10% normal goat serum (NGS; Dianova, Hamburg, Germany) or 10% normal donkey serum (NDS; Dianova, Hamburg, Germany) for 30 min at room temperature. For immunoreactivity to CK18, NOS1, ChAT, and SNAP25 cryosections underwent citrate-antigen-retrieval. Therefore, frozen sections were incubated in sodium citrate buffer (10 mM sodium citrate, 0.05% Tween 20, pH 6.0) for 45 min at 4°C. Afterwards sections were immersed in the same sodium citrate buffer for 5 or 10 min at 100°C. After three rinses for 5 min in 1× PBS, cryosections were blocked in 0.3% Triton X-100 in 1× PBS containing either 10% NGS or 10% NDS for 30 min at room temperature. For single- and double-labeling experiments, primary antibodies were diluted in 0.3% Triton X-100 in 1× PBS containing either 10% NGS or 10% NDS. Antibodies were used in the following dilutions: mouse anti-cytokeratin18 (61028; Progen Biotechnik, Heidelberg, Germany) 1:80; rabbit anti-TRPM5 serum73 [purified antibody (AB-321) described in Kaske et al. (2007)] 1:500; rabbit anti-PLCβ2 (sc-206, Santa Cruz Biotechnology, SantaCruz, CA, USA) 1:100; rabbit anti-gustducin [described in Sothilingam et al. (2011)] 1:300; rabbit anti-NOS1 (sc-8309, SantaCruz Biotechnology, SantaCruz, CA, USA) 1:50; rat anti-NCAM [“anti-BSP-2” described in Hirn et al. (1983)] 1:50; rabbit anti-GFP (Invitrogen, Karlsruhe, Germany) 1:500; goat anti-ChAT (AB144P, Millipore, Temecula, CA, USA) 1:50/1:100; mouse anti-SNAP25 (ab-24737, Abcam, Cambridge, UK) 1:100.

Blocked sections were incubated with the diluted primary antibodies overnight at 4°C. After washing in 1× PBS, the bound primary antibodies were visualized using appropriate secondary antibodies conjugated to Alexa 488 or Alexa 568 (Invitrogen, Karlsruhe, Germany, 1:500) diluted in 1× PBS with 0.3% Triton X-100 containing either 10% NGS or 10% NDS for 2 h at room temperature. After three rinses for 5 min in 1× PBS, the sections were counterstained with 4,6-diamidino-2-phenylindole (DAPI; 1 μg/mL, Sigma Aldrich, Schnelldorf, Germany) for 3 min at room temperature, rinsed with bidest, and finally mounted in MOWIOL. No immunoreactivity could be observed when the primary antibodies were omitted.

### Microscopy and photography

Digital photographs of mouse stomach were taken with an Epson standard digital camera (Epson, Meerbusch, Germany). Immunohistochemical staining was documented by using a Zeiss Axiophot microscope (Carl Zeiss MicroImaging, Jena, Germany). Images were captured using a “Sensi-Cam” CCD-camera (PCOimaging, Kelheim, Germany). For confocal microscopy of immunohistochemical staining experiments, a Zeiss LSM 510 META system was used. *In situ* hybridizations were photographed by using a Zeiss Axioskop 2 with an AxioCam MRc5 (Carl Zeiss MicroImaging, Göttingen, Germany). Images were adjusted for contrast in AxioVision LE Rel. 4.3 (Carl Zeiss MicroImaging, Jena, Germany) and arranged in PowerPoint (Microsoft) and Adobe Photoshop (Adobe Systems, San Jose, CA, USA).

### Cell quantification

On cross sections of the murine gastric mucosa cells stained with either CK18, TRPM5, or gustducin antibodies were counted. The total number of cells was determined by counting cell nuclei stained with DAPI. Several areas (*n* = 15) of corpus epithelium from the “gastric groove” (size 150 × 50 μm or 100 × 50 μm, respectively) were analyzed. Cell numbers were determined in areas at the greater curvature as well as in areas at the esophagal orifice. Only cells at the most apical layer facing the “gastric groove” were included in the quantitative analyses. For cell quantification, values are given as mean ± SD.

## Results

### Arrangement of TRPM5-expressing brush cells along the “gastric groove”

**Figure 1:**
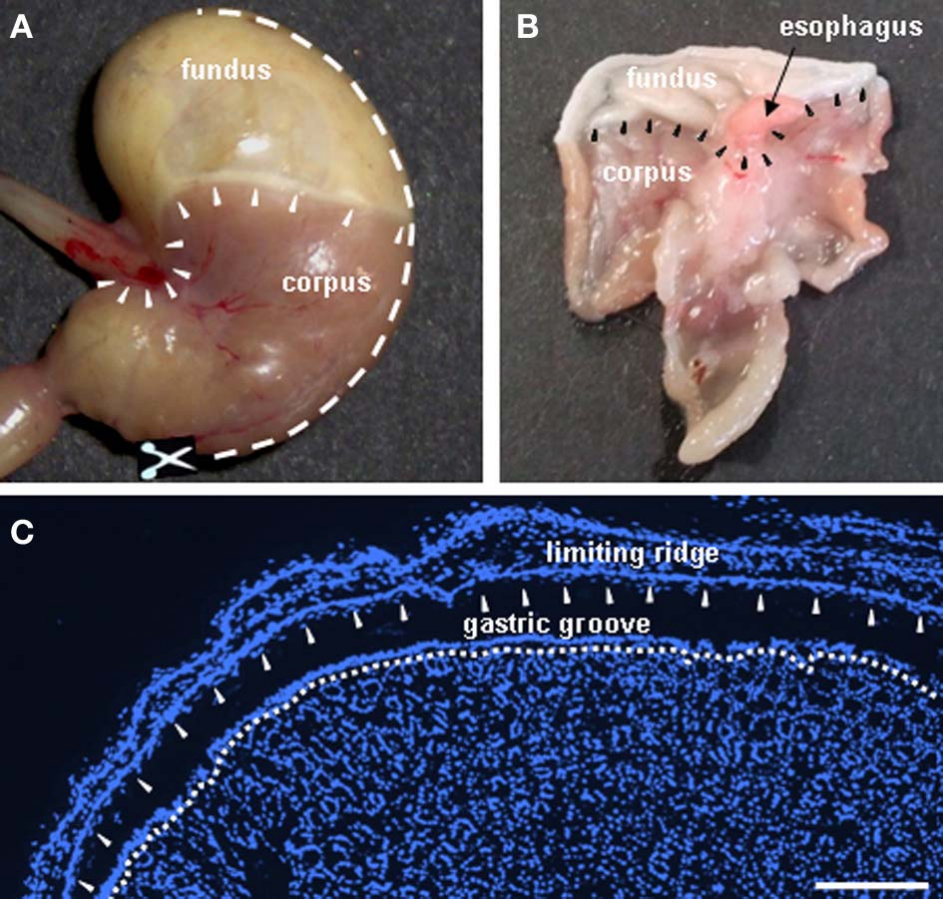
**Anatomy of the murine stomach: sections were made through the gastric mucosa encompassing the “limiting ridge” which covers the adjoining corporal mucosa. (A)** Digital photograph showing a view onto the mouse stomach. *Arrowheads* display the course of the “limiting ridge” around the esophagal orifice and along the boundary between fundus and corpus. The *white broken line* denotes sectioning; to open the stomach, the bottom of the greater curvature was dissected along the greater curvature to the fundic compartment **(B)**. After opening the stomach along the greater curvature, the gastric mucosa was embedded flatly as demonstrated in the digital photograph and sections were made from the luminal side of the gastric mucosa, resulting in cross sectioned mucosal glands and the prolonged arrangement of the “limiting ridge” accompanying the corpus tissue in a small distance **(C)**. *Arrowheads* depict the course of the “limiting ridge.” **(C)** Cryosection demonstrating the fundus/corpus boundary of the gastric mucosa. The “limiting ridge,” denoted by the *arrowheads* appears as a tissue strip covering the underlying apical corpus mucosa which forms the distal wall of the “gastric groove.” The *white broken line* depicts the most apical cell layer of the corpus mucosa facing the lumen of the “gastric groove.” Sections were counterstained with DAPI (*blue*). *Scale bar:* **(C)** = 200 μm.

**Figure 2:**
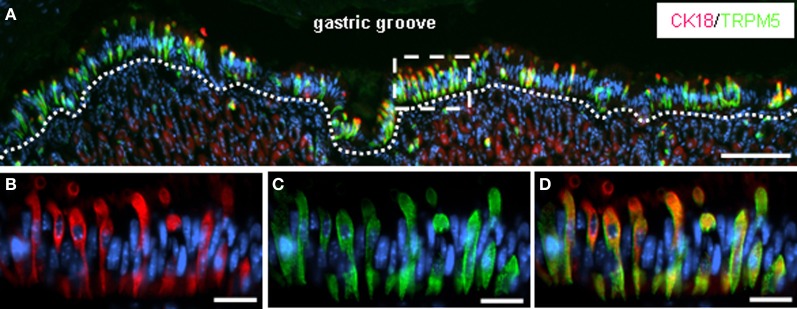
**CK18-positive brush cells lining the boundary between fundus and corpus are immunoreactive for TRPM5. (A)** Double-immunolabeling of cryosections through the gastric mucosa at the junction between fundus and corpus with specific antibodies against CK18 *(red)* and TRPM5 *(green)*. The overview of the apical corpus mucosa adjoining the fundus epithelium shows a strikingly high number of CK18-/TRPM5-immunoreactive brush cells all along the course of the “limiting ridge” facing the lumen of the “gastric groove.” The *white dotted line* defines the most apical cell layer of the corpus mucosa comprising the clustered brush cells. **(B–D)** Higher magnification of the *boxed area* in **(A)** shows CK18- **(B)** and TRPM5- **(C)** immunoreactive cells exhibiting the typical elongated cylindrical morphology of brush cells. The apical cellular processes reach the lumen of the “gastric groove.” All cells are costained for CK18 and TRPM5, as can be seen in the overlay **(D)**. Sections were counterstained with DAPI *(blue)*. *Scale bars*: **(A)** = 100 μm; **(B–D)** = 20 μm.

In previous studies it has been shown that a strikingly high number of putative chemosensory cells are located at the boundary between fundus and corpus mucosa, underneath a tissue fold called “limiting ridge” (Höfer et al., 1996; Hass et al., 2007). However, it is still unclear how these cells are arranged along the course of the tissue fold. This is mainly due to the fact that cross sections through the gastric mucosa usually only represent short segments of the “limiting ridge” and the underlying corpus mucosa. If and how these cells are positioned along the entire length of the tissue fold, which encircles the stomach in its whole circumference, is difficult to clarify. Therefore, a sectioning technique was established which enabled us to analyze the corpus surface epithelial cells facing the “gastric groove” over a long distance (Figure 1). Immunohistochemical staining of the sections with an antibody for cytokeratin 18, a marker of brush cells (Höfer and Drenckhahn, 1996), visualized a high number of immunoreactive cells, which appeared to be mostly organized in clusters (Figure 2A). These cells were positioned all along the “gastric groove” bordering the whole length of the corpus surface epithelium. Costainings for cytokeratin 18 and TRPM5 revealed that apparently all brush cells expressed the transient receptor potential ion channel TRPM5 (Hofmann et al., 2003), which is involved in gustatory signal transduction (Figures 2A–D).

### Coexpression of gustatory transduction elements in cells of the “gastric groove”

**Figure 3:**
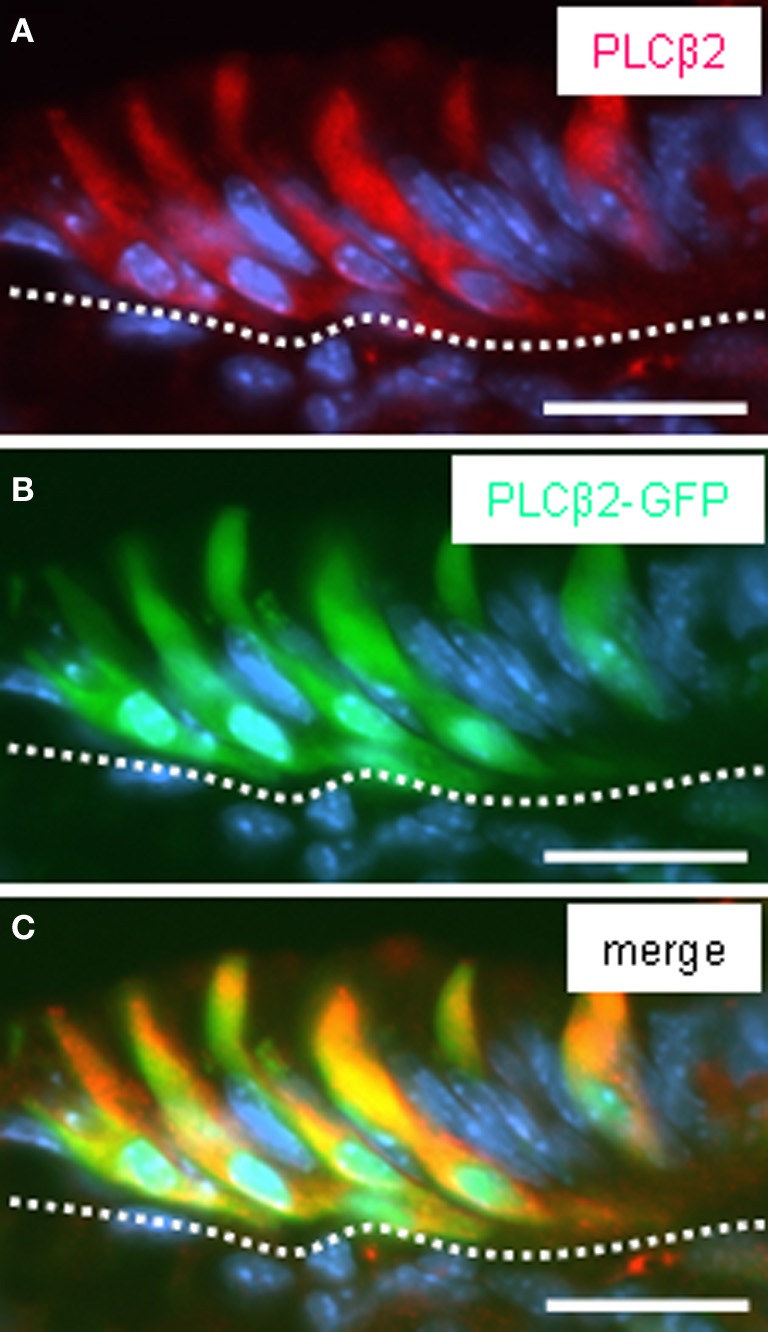
**Pattern of GFP fluorescence in transgenic PLCβ2 promoter-GFP mice correlates with endogenous PLCβ2 expression in cells of the corpus mucosa adjacent to the fundus compartment. (A–C)** Analysis of cross sections through the fundus/corpus transition zone of PLCβ 2 promoter-GFP transgenic mice. PLCβ 2-immunostaining *(red)* **(A)** reveals a cluster of elongated cells located in the most apical layer of the corpus mucosa (denoted by the *white dotted line*) adjoining the fundus epithelium. Intrinsically green fluorescent cells **(B)** are located in the same regions within the mucosa and display the morphology of cells visualized by the PLCβ 2 antibody. Overlay **(C)** of **(A)** and **(B)** clearly demonstrates the coexpression of PLCβ2 and GFP in a subset of cells. The cells exhibit very long apical processes extending into the lumen of the “gastric groove.” Sections were counterstained with DAPI (*blue*). *Scale bars:* **(A–C)** = 20 μm.

**Figure 4:**
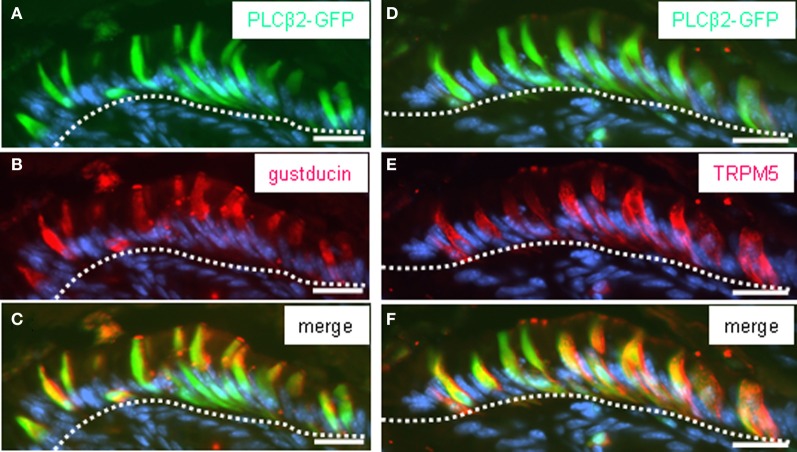
**PLCβ2-GFP-expressing cells of the corpus mucosa underneath the “limiting ridge” are immunoreactive for gustducin and TRPM5. (A–C)** Distribution pattern of gustducin *(red*) was assessed on cross sections through the corpus mucosa adjoining the fundus tissue of PLCβ 2 promoter-GFP transgenic mice. The *white dotted line* borders the most apical cell layer from the residual corpus mucosa. Cluster of cells immunostained for gustducin **(B)** underneath the “limiting ridge” exhibit an elongated cylindrical morphology and strongly labeled apical microvilli tufts. Pattern of intrinsic GFP fluorescence **(A)** is reminiscent of the labeling pattern obtained with the gustducin antibody **(B)**. Overlay **(C)** of **(A)** and **(B)** shows that a subpopulation of cells express both gustducin and GFP. **(D–F)** Consecutive sections to that shown in **(A–C)** stained with the TRPM5 antibody (*red*). TRPM5-immunoreactivity reveals a cluster of cells **(E)** resembling that of PLCβ2-GFP-expressing cells **(D)**, in both morphology and position, respectively. Overlay **(F)** of **(D)** and **(E)** indicates a complete overlap of GFP and TRPM5 in a subpopulation of corpus mucosal cells. Sections were counterstained with DAPI (*blue*). *Scale bars*: (**A–F**) = 20 μm.

There is some evidence from previous studies indicating that these cells express further elements, which are typical for gustatory “type II” cells, including gustducin (Höfer et al., 1996; Hass et al., 2007). To explore whether the brush cells of the “gastric groove” also express the phospholipase C-subtype PLCβ2, the key enzyme of the gustatory cascade, we have analyzed transgenic mice which express GFP under the control of the PLCβ 2 promoter (Kim et al., 2006). To verify that the expression of GFP in fact indicates the expression of PLCβ 2 in labeled cells, the pattern of GFP fluorescence was compared with immunohistochemical staining using a specific antibody for PLCβ 2 on sections through the gastric mucosa of the transgenic animals. Figure 3 shows that all PLCβ 2-immunoreactive cells were labeled by intrinsic GFP. This result demonstrates that the expression of GFP faithfully indicates the expression of PLCβ 2. This now allows to perform double-labeling experiments comparing the distribution of PLCβ 2-expressing cells in relation to that of putative chemosensory brush cells. Figure 4A shows a cluster of intensely labeled cells, visualized by their strong intrinsic GFP fluorescence. Apparently the cells which have an elongated body extend into the lumen of the “gastric groove,” reminiscent of the characteristic morphology of brush cells (Höfer and Drenckhahn, 1996). Using a specific antibody immunohistochemical visualization of gustducin (Figure 4B) on the same tissue sections displayed a complete overlap of green fluorescent protein and gustducin (Figure 4C). Interestingly, gustducin-immunostaining was particular strong at the most apical region of the cells, which apparently represents the apical microvillar tuft of the brush cells; such a distribution pattern for gustducin was also shown for taste cells (Takami et al., 1994). This observation deviates from results of a previous study where PLCβ 2-immunoreactivity could not be visualized in gustducin-expressing cells of the “gastric groove” (Hass et al., 2007). This discrepancy is probably due to the facts that, encouraged by the endogenous fluorescence visible in PLCβ2 promoter-GFP mice during the course of the study, we have optimized technical parameters (fixation, incubation, visualization) for the PLCβ 2-antibody.

In previous *in situ* hybridization studies, we have visualized mRNA for TRPM5 in some cells located beneath the “limiting ridge.” It was proposed that they may correspond to gustducin-expressing cells, in the same region on consecutive sections (Hass et al., 2007). The availability of PLCβ 2 promoter-GFP transgenic mice and specific TRPM5 antibody now allowed us to visualize TRPM5 protein on tissue sections. As shown in Figures 4D–F, TRPM5-immunostaining visualized a cluster of elongated cells, which also showed an intense intrinsic GFP fluorescence. These findings demonstrate for the first time that the cells arranged at the distal wall of the “gastric groove” comprise gustducin, PLCβ 2 as well as TRPM5, i. e., the important elements of the gustatory transduction cascade. Determining the number of labeled cells on cross sections, stained with antibodies for CK18, TRPM5, and gustducin resulted in very similar values for each marker supporting the notion that most of the cells express all three marker proteins. Furthermore, the results reflected the striking high proportion of putative chemosensory cells in the mucosa underneath the “limiting ridge” (Table 1).

**Table 1:** **Percentage of immunoreactive cells underneath the “limiting ridge” at corpus/fundus transition and esophagal orifice**.

	**Corpus/fundus transition**	**Esophagal orifice**
CK18	28.3 ± 13.1	17.6 ± 8.3
TRPM5	28.5 ± 12.8	19.2 ± 9.2
Gustducin	23.4 ± 8.5	17.7 ± 9.4

Cross sections through the corpus mucosa were stained immunohistochemically with antibodies for CK18, TRPM5, and gustducin. Numbers of immunoreactive cells were counted in defined fields (corpus/fundus transition: *n* = *16*; esophagal orifice: *n* = *15*) of the most proximal corpus mucosa underneath the “limiting ridge” at the greater curvature, separately to the esophagal orifice. To define the percentage of immunolabeled cells per fields, the total number of DAPI-labeled nuclei was counted. For cell quantification values are given as mean ± SD.

### Intercellular signaling capacity of brush cells of the “gastric groove”

**Figure 5:**
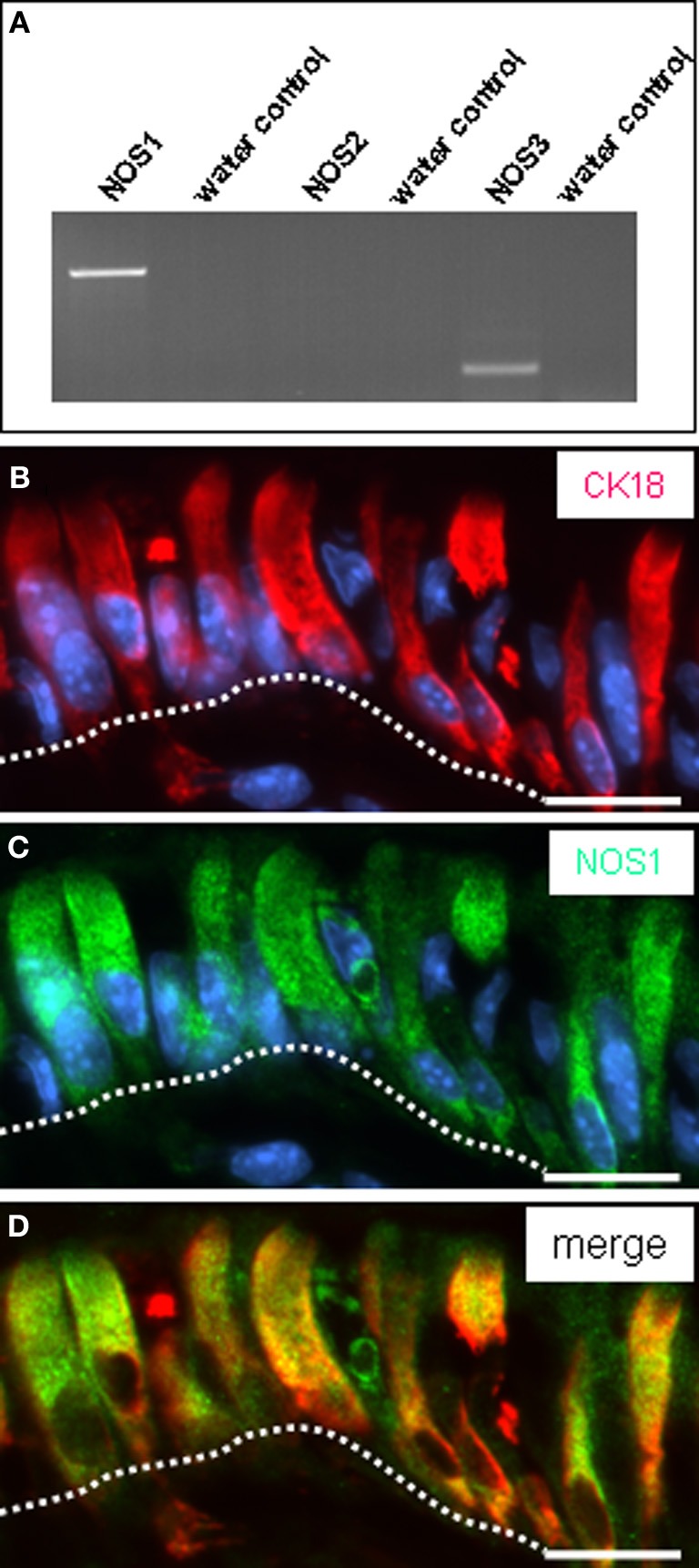
**Expression of NO-synthase 1 (NOS1) in brush cells. (A)** Identification of mRNA for NOS isoforms in the corpus mucosa. Semi-quantitative RT-PCR experiments for NOS1 (680 bp), NOS2 (499 bp), and NOS3 (167 bp) yielded amplicons of the expected size from cDNA of tissue of the fundus/corpus transition zone. While no or rather only a weak band was obtained for the isoforms NOS2 and NOS3, a strong band was amplified for NOS1. **(B–D)** Double-immunolabeling employing antibodies against CK18 *(red)* and NOS1 *(green)* on corpus tissue sections. The NOS1 antibody **(C)** labels numerous corporal surface cells (delineated by the *white dotted line*). Co-staining with CK18 antibody reveals that all CK18-positive cells are immunoreactive for NOS1 (**D**). Sections were counterstained with DAPI (*blue*). Scale *bars*: **(B–D)** = 20 μm.

The brush cells beneath the “limiting ridge” appear to comprise the molecular equipment for sensing the chemical composition of the gastric luminal content. It is, however, unclear how such information might be transmitted onto “effector” cells. In a previous study evidence was provided indicating that some brush cells of the rat gastric cardia express nitric oxide synthase (NOS) (Kugler et al., 1994) proposing nitric oxide as gaseous transmitter. RT-PCR analyses of gastric mucosa from the “gastric groove” yielded a strong amplification product indicative for mRNA encoding the neuronal nitric oxide synthase (NOS1) isoform (Figure 5A). No or rather weak bands were obtained for the inducible (NOS2) and endothelial (NOS3) isoforms. Double-immunohistochemical staining with CK18 and NOS1 antibodies visualized epithelial cells which were all immunoreactive for both CK18 and NOS1 (Figures 5B–D).

**Figure 6:**
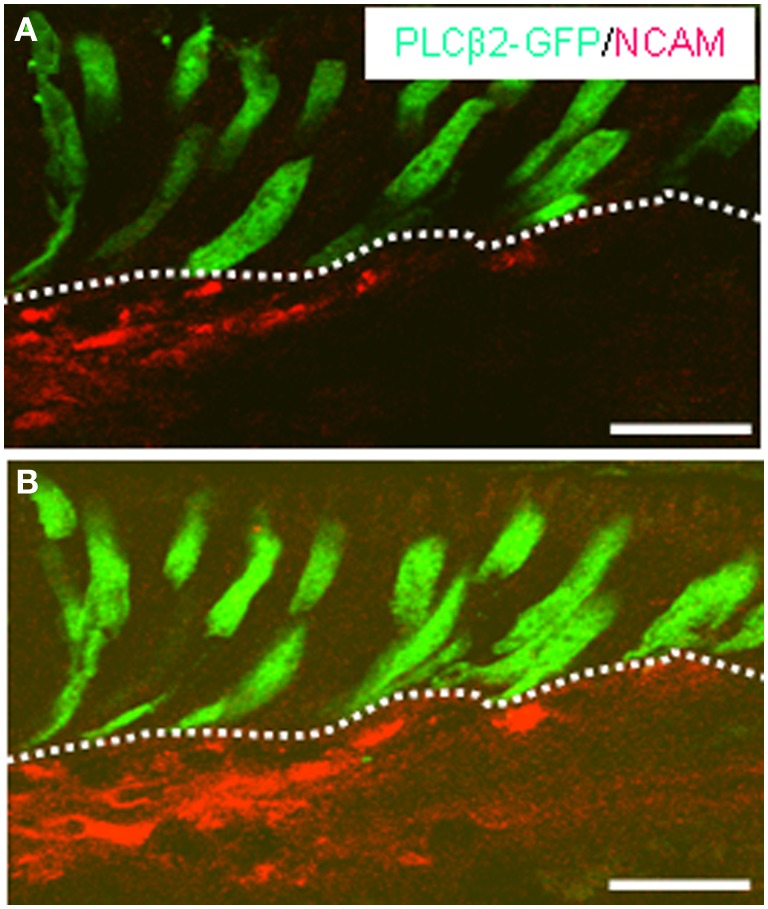
**NCAM-immunostaining displays the distribution of nerve fibers in close vicinity to GFP-expressing cells. (A,B)** Projections of confocal image stacks of cross sections through the corpus mucosa of PLCβ2 promoter-GFP transgenic mice. Immunostaining with NCAM antibody (*red*) indicates the trajectory of a dense fiber bundle meshwork near the basolateral pole of the GFP-immunoreactive (*green*) cells. The cluster of elongated GFP-positive cells are located in the most apical cell layer (bordered by the *white dotted line*) contacting with their apical processes the lumen of the groove built by the “limiting ridge.” In direct vicinity to the GFP-expressing cell cluster NCAM-positive fibers can be observed. Note the long basolateral process of these cells apparently extending toward the stained fibers. *Scale bars*: **(A,B)** = 20 μm.

It has been proposed that beside enteroendocrine cells, afferent fibers of the enteric nervous system may operate as “output” cells as they interfuse the mucosal epithelium extensively. Using a specific antibody for the neuronal marker NCAM, immunohistochemical stainings displayed a dense network of NCAM-positive fibers in close vicinity to the basolateral pole of GFP-expressing cells underneath the “limiting ridge” (Figure 6). Projections of confocal image stacks enabled us to track the course of the fibers. Figure 6B clearly illustrates that the fibers ran immediately next to the basolateral pole of the fluorescent cells. Nevertheless direct contact between the cells and the neuronal processes, as in terms of synapses, could not be identified.

**Figure 7:**
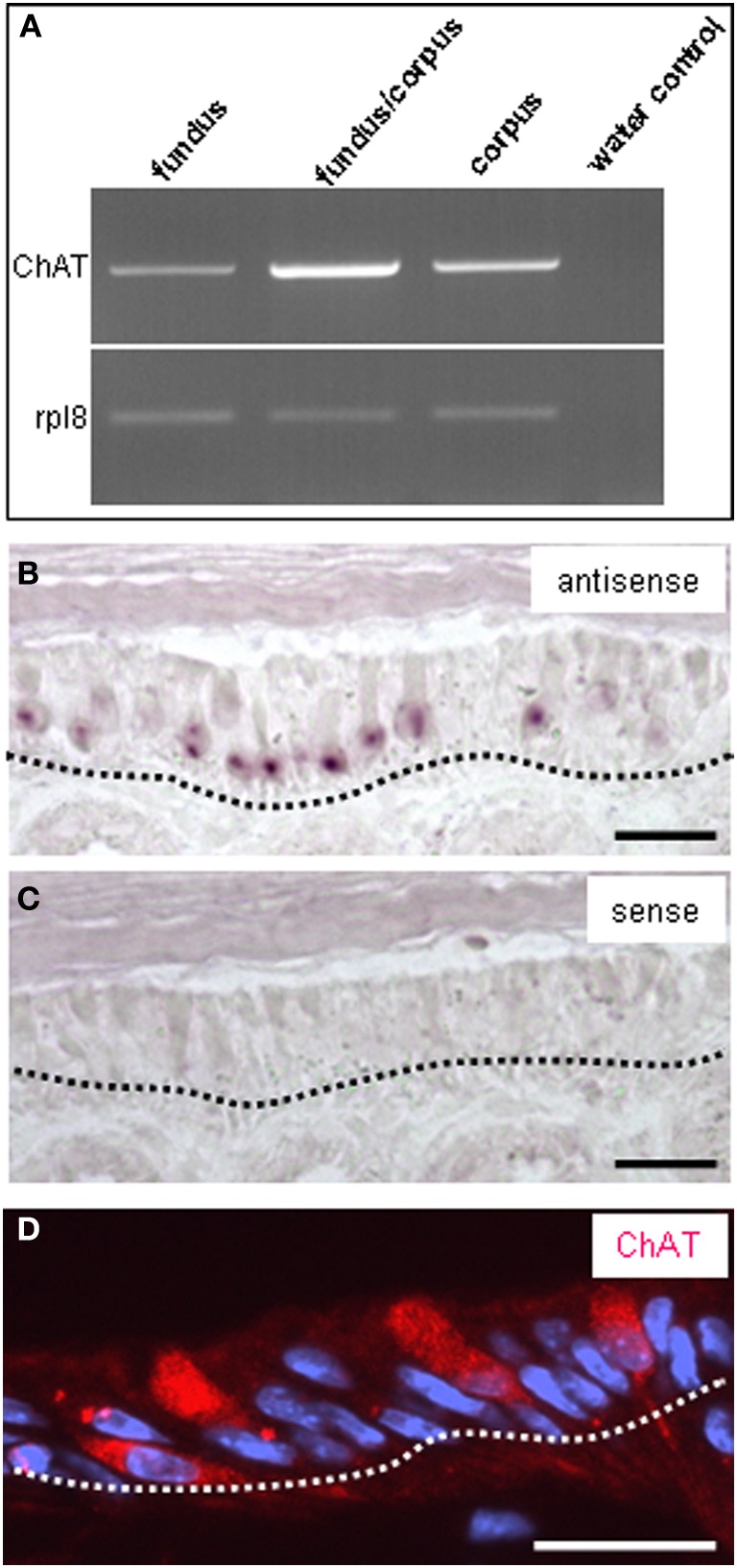
**Expression of choline acetyltransferase (ChAT) and the ribosomal protein l8 (rpl8) in the gastric mucosa. (A)** Semi quantitative RT-PCR approaches for choline acetyltransferase (1093 bp) or rpl8 (204 bp) with normalized cDNA from gastric tissue of fundus, fundus/corpus transition zone, and corpus resulted in amplicons of the expected size for each region; the strongest band was obtained for the fundus/corpus transition zone. **(B)** *In situ* hybridization experiments with an antisense riboprobe for ChAT reveals the expression pattern of the enzyme. Cluster of mRNA-expressing cells can be visualized in the most apical corpus mucosa (depicted by *black dotted line*) directly underneath the “limiting ridge.” **(C)** Control experiments with the corresponding sense riboprobe for ChAT show no staining. **(D)** Immunostaining with an antibody against choline acetyltransferase labels numerous cells in the most apical mucosa cell layer (denoted by the *white dotted line*) underneath the “limiting ridge,” reminiscent of the position of ChAT mRNA-expressing cells seen in *in situ* hybridization experiments in **(B)**. Sections were counterstained with DAPI (*blue*). *Scale bars:* **(B–D)** = 20 μm.

**Figure 8:**
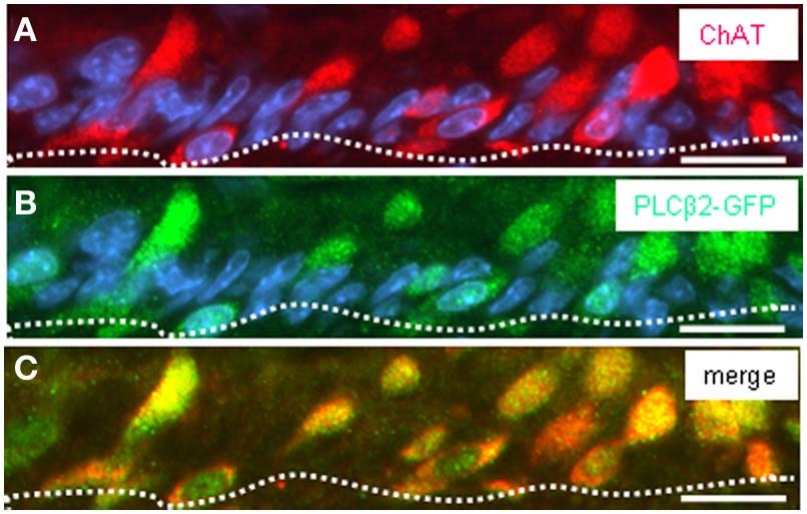
**Double-immunohistochemical staining visualizing ChAT protein in GFP-expressing cells of transgenic PLCβ2 promoter-GFP mice. (A–C)** Analysis of ChAT immunoreactivity (*red*) pattern in relation to GFP staining pattern (*green*) on cross sections through the corpus mucosa at the fundus/corpus boundary of transgenic PLCβ2 promoter-GFP mice. **(A)** Numerous cells of the most apical mucosal cell layer (defined by the *white dotted line*) are immunoreactive for ChAT. For several cells, the cross sectioned elongated apical pole appears as round-shaped structure. Staining with the GFP antibody **(B)** reveals many immunoreactive cells similar to the ChAT distribution pattern in **(A)**. Overlay **(C)** of **(A)** and **(B)** reveals coexpression of ChAT and GFP in a subset of corpus mucosal cells. Sections were counterstained with DAPI (*blue*). *Scale bars*: **(A–C)** = 20 μm.

**Figure 9:**
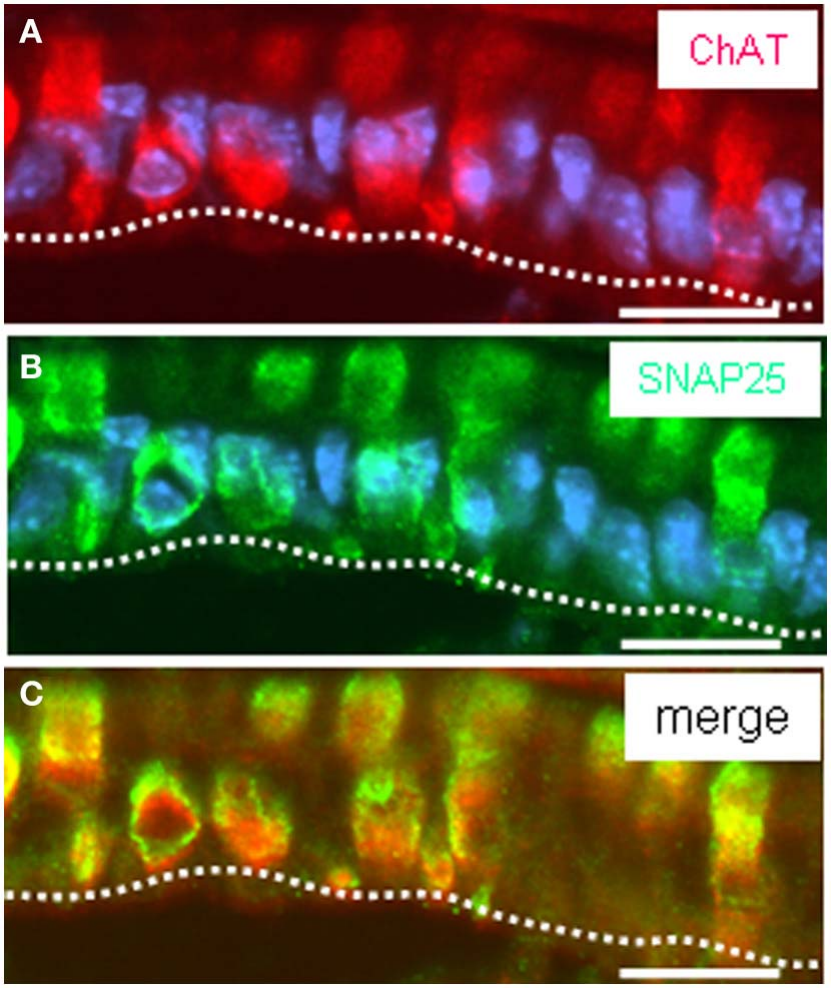
**Acetylcholine synthesizing cells express the vesicular protein SNAP25. (A–C)** Assessment of SNAP25 and ChAT-immunostaining on cross sections through the corpus mucosa at fundus/corpus boundary using specific antibodies against ChAT (*red*) and SNAP25 (*green*). ChAT-immunostaining visualized clustered ChAT-expressing cells extending into the “gastric groove” built by the “limiting ridge” **(A)**. The SNAP25 antibody reveals a pattern of immunostaining **(B)** reminiscent of that of ChAT **(A)**. Overlay **(C)** of **(A)** and **(B)** clearly exposes coexpression of ChAT and SNAP25 in all stained corpus mucosal cells underneath the “limiting ridge.” Sections were counterstained with DAPI (*blue*). *Scale bars*: **(A–C)** = 20 μm.

Thus, a paracrine interaction between the mucosal cells and the nerve fibers seemed plausible, as was previously demonstrated for solitary chemosensory cells of the vomeronasal organ (Ogura et al., 2010); these cells were found to express ChAT, the enzyme for synthesizing acetylcholine. To determine whether the putative chemosensory cells of the “gastric groove” may also be cholinergic, we investigated the gastric mucosa for an expression of ChAT. Semi-quantitative RT-PCR analyses of different mucosal regions indicated that the boundary region between fundus and corpus epithelium which includes the “limiting ridge” contained a high level of mRNA for ChAT compared to fundus and corpus tissue (Figure 7A). *In situ* hybridization experiments revealed numerous ChAT specific hybridization signals in the most apical cell layer of the corpus mucosa directly beneath the “limiting ridge” (Figure 7B). This result was confirmed in immunohistochemical studies using a specific ChAT antibody. As demonstrated in Figure 7D labeled cells were visualized in same corpus mucosa region. Double-labeling experiments on tissue sections from the PLCβ 2 promoter-GFP mice (Figure 8) demonstrated that ChAT-immunoreactivity pattern completely overlapped with the endogenous fluorescence. Thus, putative chemosensory brush cells are capable to generate the transmitter acetylcholine. In cholinergic synapses proteins of the SNARE complex are essential for the release of ACh. Exemplarily, we focused on one experiment using a specific antibody for SNAP25, an important member of the SNARE complex, resulting in numerous labeled cells (Figure 9B), as seen for the ChAT-immunostaining (Figure 9A). Costainings with the ChAT antibody resulted in a complete overlap of the labeling indicating expression of SNAP25 in ChAT-positive cells (Figure 9C; the more round shaped appearance is due to an angular section plane). The results point to an exocytotic release of acetylcholine from putative chemosensory cells in the “gastric groove.” This observation is in line with the extensive cholinergic circuitries in the enteric nervous system including nicotinic and muscarinic receptors [reviewed by Harrington et al. (2010)].

## Discussion

A strikingly high number of brush cells, CK18-positive cells, appear to exist at the esophagal orifice as well as at the junction between fundus and corpus of the mouse and rat stomach, the so called “limiting ridge” (Wattel and Geuze, 1978; Höfer and Drenckhahn, 1992; Luciano and Reale, 1992; Höfer and Drenckhahn, 1996; Hass et al., 2007). In this study a newly established sectioning approach allowed visualization of the arrangement of these cells along a relatively long segment and revealed a palisade-like assembly of these brush cells all along the course of the “limiting ridge.” It turned out that at the “gastric groove” at the fundus/corpus boundary about 30% of the cells were brush cells (Table 1); this number confirms previous finding of Akimori et al. (2011) exceeding the percentage in other regions of the GI tract by more than 10 times. So far, the characteristic tissue fold called “limiting ridge” has been described only for rodents (Wattel and Geuze, 1978; Luciano and Reale, 1992); however, it cannot be excluded that such an arrangement may also occur in other species, especially in those with a fundus that has structural features which are strikingly different from other gastric compartments. Based on their shape and position, it has been proposed that brush cells may be candidate chemosensory cells. Comparing the percentage of CK18-positive brush cells with TRPM5 and gustducin-immunoreactive cells revealed that the number of labeled cells was similiar for all three marker proteins. These findings substantiate the notion that a large portion of brush cells at the “gastric groove” may express the gustatory marker TRPM5 and gustducin. The small discrepancies between the numbers for CK18-, TRPM5-, and gustducin-immunoreactive cells are probably due to technical limitations. The ability to stain tissue sections from PLCβ 2 promoter-GFP transgenic mice for TRPM5 and gustducin revealed a complete overlap of TRPM5 and gustducin with PLCβ 2 in cells at the “gastric groove,” indicating that a remarkably high number of cells at the “gastric groove” express all three of these molecular elements of the gustatory signaling cascade. This finding differs from the result of a previous study where PLCβ 2-immunoreactivity was not visualized in gustducin-positive brush cells (Hass et al., 2007). This deviation can most likely be explained by the fact that over the last 5 years the technical conditions and procedures of immunohistochemical experiments analyzing the gastric mucosa, such as fixation, antibody concentrations, and incubation times, were considerably improved. Furthermore it was found that for an optimal staining intensity of the current immunohistochemical procedures the PLCβ 2-antibody required a fixation of the tissue for a rather narrow time interval. It is probably due to these reasons that in this study we were able to visualize PLCβ 2-expression in the CK18-/gustducin-positive cells at the “gastric groove,” which was not seen in the prior study by Hass et al. (2007). Thus, the CK18-/gustducin-expressing cells located as a palisade-like band at the “gastric groove” are endowed with several taste-like elements including PLCβ 2. The observation that brush cells comprise all transduction elements of gustatory sensory cells which are responsive for the detection of bitter, sweet, and umami stimuli substantiates previous speculations about a possible role of brush cells as chemosensory cells, capable to monitor luminal content. Although the receptor types which may render these cells responsive to nutrients of the ingested food are not identified yet, it is interesting to note that at least some of the cells at the “limiting ridge” express T1R3, a subunit of the sugar and amino acid receptor (Hass et al., 2010). For solitary chemosensory cells in other tissues the expression of distinct bitter receptors has recently been described (Finger et al., 2003); although so far all attempts to visualize the expression of bitter receptors remained unsuccessful, it is conceivable that the putative sensory cells at the “gastric groove” may contribute to the detection of potentially harmful food ingredients which often taste bitter.

For solitary chemosensory cells in the nose or the trachea there is some evidence suggesting that they may interact with nerve fibers (Tizzano et al., 2010; Krasteva et al., 2011). Since the putative chemosensory brush cells at the “gastric groove” are apparently not enteroendocrine cells, i.e., releasing signaling molecules into the blood circulation, it seems possible that they interact with nerve fibers of the enteric nervous system. Our finding that a dense network of NCAM-positive fibers is located at the basolateral pole of the cells (Figure 6) supports this notion. The finding that most of the putative chemosensory cells express ChAT, the enzyme for synthesizing acetylcholine may be indicative for a cholinergic communication. In fact, acetylcholine is known as an important transmitter in the extensive cholinergic circuits of the enteric nervous system. However, the evidence for distinct acetylcholine receptors in the gastric system is mainly based on electrophysiological and pharmacological experiments rather than histochemical studies (reviewed by Harrington et al., 2010); thus the knowledge about the localization of the receptor proteins is still very limited. So, it is currently impossible to determine whether a cholinergic communication may exist between the ChAT-positive brush cells and the intrinsic primary afferent neurons (IPANs) (Furness et al., 2004). The notion that the capacity of brush cells to synthesize acetylcholine may be functionally relevant was supported by the finding that these cells also express the vesicular protein SNAP25, an important element of the SNARE complex, suggesting an exocytotic release of acetylcholine (Foran et al., 2003). The finding that the cells also express NOS parallels previous findings for brush cells of the cardia region (Kugler et al., 1994). The short-lived, gaseous molecule nitric oxide can only affect nearby cells, thus it could possibly act as signaling molecule between brush cells and enteroendocrine cells, since numerous ghrelin-cells as well as serotonin-cells are located in the vicinity of the “gastric groove” (Hass et al., 2007). These results indicate that at least the majority of brush cells have the enzymatic capacity to generate two types of signaling molecules, nitric oxide and acetylcholine. Thereby, the brush cells may be able to communicate information about the chymus composition onto adjacent cells, which could be endocrine cells as well as enteric nerve fibers from afferent neurons, interneurons, or motoneurons. The present findings substantiate the hypothesis that brush cells in the gastric mucosa may operate as chemosensory cells which monitor the composition of ingested food and convey the information onto regulatory systems which adjust gastric activities according to the luminal content. Ongoing studies are concentrating on the identification of receptor types which are capable for sensing the constituents of the gastric chyme and to trigger the signaling processes in the gastric sensory cells.

### Conflict of interest statement

The authors declare that the research was conducted in the absence of any commercial or financial relationships that could be construed as a potential conflict of interest.

## Abbreviations

ChAT: Choline acetyltransferase
CK18: Cytokeratin 18
DAPI: 4,6-diamidino-2-phenylindole
GI: Gastrointestinal
NCAM: Neural cell adhesion molecule
NO: Nitric oxide
NOS: Nitric oxide synthase
PLCβ2: Phospholipase C beta2
T1R3: Taste receptor type 1, member 3
TRPM5: Transient receptor potential cation channel, subfamily M, member 5.
